# Treatment of Colic with Chloride of Barium1Translated by Otto Noack, Veterinarian, Reading, Pa.

**Published:** 1896-07

**Authors:** 


TREATMENT OF COLIC WITH CHLORIDE OF BARIUM.[1] Referring further to the treatment of colic in horses with the salts of arecolin and chloride of barium, further experiments with this

[1] Translated by Otto Noack, Veterinarian, Reading, Pa.

latter agent on a number of cases large enough to satisfy myself of its claimed therapeutic result as announced by Dieckerhoff. At the meeting on December 12th Mr. Moller-can reported three unfortunate cases out of a total of thirty-eight injections, and in these cases it is necessary to acknowledge the product injected into the blood seems to have caused the deaths.

Mr. Ries, of Luxembürg, an old pupil of the College of Alfort, has left it to me to report to you a new case of intoxication from a small dose of chloride of barium. The following is the note of Mr. Ries: "Having read the work of Dieckerhoff on using the chloride of barium for treatment of colics, I have tried to instruct myself on the question to know to what extent this salt is used in medicine. It figures in the old German *Pharmacopœia* among the number of inoffensive or little energetic medicaments. In the last edition of said *Pharmacopœia* it is not mentioned at all. Hager (1876) said the authors of the *Pharmacopœia Germanica* did not know the numerous deadly intoxications caused by this salt; he shows plainly the *Pharmacopœia* indicates too strong doses. The use of chloride of barium has been in preparations for the destruction of mice and rats. Hager adds, chloride of barium belongs to the group of the acrid toxins. This agent produces death by cardiac syncope. It was used against scrofula, in certain affections of the eyes, in chronic orchitis. To-day it is completely abandoned.

"This is what I learned about this salt since you have made your communication to the Société Centrale de Médecine Vétérinaire. This communication confirmed the remarkable tolerance of the organism of the horse in regard to this heart-poison, even when the chloride was administered in intravenous injection.

"On December 6th, 3.30 P.M., a gelding of heavy build, seven years old, was brought to me affected with colic. This horse had never been sick. He had just travelled about six and a half English miles at a walk, pulling a cart, not heavily loaded. He had been used immediately after his meal. Arrived at his destination he was placed in the stable of the inn. Half an hour later the proprietor noticed the colic, and had the horse immediately sent to me.

"*Symptoms.*—Patient lays down carefully; don't roll much; the abdomen is not distended; the conjunctiva normal in aspect; heart and pulse functions regular, only a slight acceleration at the moment of the attack.

"*Diagnosis.*—Acute indigestion.

"*Treatment.*—A bottle of coffee is immediately administered. At four o'clock he gets a calming drench (tincture of opium, tincture of asafœtida). At 4.30 P.M. I give an injection of fifty centigrammes of morphine. The patient lays down and sleeps three-quarters of an hour. At five o'clock the colic returns as before. The general state has remained the same. Eserine is indicated; patient by no means exhausted, not even tired. Pulse remains normal. Hesitated to try the chloride of barium because of mistrust one has in using medicaments which we do not know. I recommend to the pharmacist to boil and filter the solution. During this time I prepare the Pravaz syringe as for vaccinations (new piston, the other parts are boiled and perfectly cleansed). Not to neglect any precaution I shave the skin at the point where the injection has to be made and wash it with carbolized water. The empty needle had produced a small jet of blood. The syringe did not contain one air-bubble. I injected fifty centigrammes of chloride of barium in solution. At the same time I draw the needle back, lay down the instruments, the patient staggers, totters, tumbles down behind, falls like a mast, and dies almost immediately. I had sustained artificial respiration with the assistance of a helper. Under the influence of the methodical compressions of the thorax, of the rising and stretching of the upturned front leg, the air could be heard entering and leaving the thoracic cavity, and that lasting a quarter of an hour. Life was extinct.

"*Autopsy.*—Overloading of stomach without a rent in the walls. Cavities of the heart filled with thick blood-clots.

"Whether to attribute this death to a small dose of chloride, to the preliminary administration of morphine, to the continuance of colic, to a peculiar state of the heart, to an abnormal sensitiveness of the patient, I do not know."

I would repeat that during the three months which have preceded my first communication we had treated in Alfort thirty-two horses affected with colic, varying the doses according to the weight of the subjects and the nature of colic, without producing a case of deadly intoxication; but none of the sick ones had exhibited alarming conditions. Mr. Almy and myself continued this medication, observing all the time the indication, injecting only small doses to sick ones showing grave circulatory troubles. Some unfortunate facts which I have mentioned compelled us to act in this way; but it was necessary for one part of these sick ones—for those which were brought at night and have been treated by the students on duty—to obey this indication. However, up to the present time we have not had a case of death attributable to the chloride of barium, and have been well pleased with the same. In the number of January 3d the *Berliner Thierärztliche Wochenschrift* records the results of the use of the chloride of barium at the college at Berlin for the last four months of 1895. Out of one hundred and thirty-nine horses affected with colic and treated with this agent, twelve have died (8.6 per cent.). In seven torsion of the colon was found; in two volvulus of the small intestines; in one strangulation of the small intestines with the epiploon; in another a strangulation of the intestine in the hiatus of Winslow; another with lesions of acute entero-peritonitis. Not one death through the action of chloride of barium. According to this new note, Brass insists upon the importance of the dosage. If the heart is already badly affected, if the pulse is much accelerated and feeble, it is necessary to keep up small injections, half of the ordinary doses. He claims that the small doses of chloride of barium produce more rapid and more wholesome effects than the other therapeutic agents which are warmly advocated at the present time.—CADIOT, in *Recueil de Médecine Vétérinaire, Bulletin de la Société Centrale de Médecine Vétérinaire, Meeting February* 27, 1896.

